# Light-Induced Dynamic Holography

**DOI:** 10.3390/mi13020297

**Published:** 2022-02-14

**Authors:** Daniele Eugenio Lucchetta, Andrea Di Donato, Melania Paturzo, Gautam Singh, Riccardo Castagna

**Affiliations:** 1 Dipartimento SIMAU, Università Politecnica delle Marche, Via Brecce Bianche, 60131 Ancona, Italy; 2 URT-CNR, Università di Camerino (UNICAM), Polo di Chimica, Via Sant’Agostino, 1, 62032 Camerino, Italy; 3 CNR ISASI, Via Campi Flegrei 34, 80078 Pozzuoli, Italy; m.paturzo@isasi.cnr.it; 4 Department of Applied Physics, Amity Institute of Applied Sciences, Amity University, Uttar Pradesh, Noida 201313, India; gautsingh@gmail.com; 5 Dipartimento DII, Università Politecnica delle Marche, Via Brecce Bianche, 60131 Ancona, Italy; 6 CNR, Institute of Heritage Science, Via Madonna del Piano, 50019 Sesto Fiorentino, Italy

**Keywords:** holography, photomobile polymer, all-optical control, photophobicity

## Abstract

Holographic photomobile polymers (H-PMP) are a novel class of photomobile materials in which holograms can be optically recorded. They can be used in a large variety of applications, including optical switches and color selectors. In this work, we show one of the most important properties of the photomobile film, which is the photophobicity of the unpolymerized parts of the photomobile mixture. In order to investigate this property, we recorded a transmission phase grating on an H-PMP film, and used a different experimental technique to measure the diffraction efficiency, surface tension, and mixture properties. The results allowed for a better understanding of the mechanism of the light-controlled bending observed in these compounds.

## 1. Introduction

High-resolution transmission and reflection holographic gratings written in polymeric materials are known and investigated for a long time [1,2], and cover a large variety of applications, ranging from high density data storage [3,4,5], to lasing [6,7], to super-fast holographic displays [8]. More recently, all optically addressable holographic gratings have been also widely studied. They are usually obtained in different ways and show peculiar properties, such as a fast oscillation frequency [9] or the possibility to be integrated into optical circuits as all-optical switches [10,11,12,13,14,15,16,17,18,19,20]. Holographic photomobile polymers (H-PMP) are photomobile materials on which holograms can be optically recorded. The optical characteristics of the recorded hologram can be tuned by using an external pumping light. In this case, the entire film can act as a switch or a color selector [21]. Other applications are possible once the working mechanism is clearly explained. In our previous works, we have focused on the optical and morphological properties of the PMP films; here, we point out our attention on the mobility of the unpolymerized part of the PMP mixture. In previous works, we underlined the possibility of moving the polymer by using a laser beam, due to the conversion of energy to mechanical work [22,23,24,25]. Here, the keyword is photophobicity, which is the tendency of the unpolymerized mixture to escape from the light when illuminated [26]. This property is a peculiarity of our PMP film and is directly responsible of its unique mechanical and optical properties. In order to investigate the properties of our materials, we optically recorded a phase transmission grating on a H-PMP film and measured its diffraction efficiency and the surface tension of the unpolymerized part. We also provided suggestions about the material properties and the underlying nature of working mechanism.

## 2. Materials and Methods

### 2.1. Materials

2,3-bornanedione (CQ), tri-phenyl-o-methane-triglicidyl ether (TPMTGE), dipentaerythritol monohydroxypentaacrylate (DPMHPA), lead (IV) tetra-acetate, para-amino-phenol (4-AP), phenyl-(2,4,6-trimethylbenzoyl) phosphoryl]-(2,4,6-trimethylphenyl) methanone (PTPTM) and N-Vinylbutyrolactam (NVP) came from Sigma Aldrich; lead(IV) oxide (PbO_2_) is freshly prepared by the hydrolysis of lead(IV) acetate.

### 2.2. H-PMP Mixture Preparation

The mixture is prepared starting from the recipe indicated in [26] to which the epoxide-monomer TPMTGE and the photo-initiator 2,3-bornane-dione (CQ) are furthermore added. Chemicals: TPMTGE (0.1 mmol):DPMHPA (1 mmol):NVP (7 mmol):4-AP (1 mmol):PbO_2_ (0.5 mmol); photo-initiators: CQ (2% *w*/*w*), PTPTM (2% *w*/*w*). Firstly, a mixture 1 is prepared as follows: DPMPHA is mixed with photoinitiators (CQ and PTPTM) under magnetic stirring at room temperature for 24 h. Then, TPMTGE is added (by heating it at 90 °C, TPMPTGE is in a low viscous form and it can be easily handled). The mixture is left under mechanical stirring until a pale yellow color is obtained. Separately, a mixture 2 is prepared as follows: NVP, 4-AP and PbO_2_ are mixed together in aerobic conditions at room temperature in darkness under magnetic stirring for 7 days. After that, the mixture is left at rest at room temperature in darkness for additional 7 days. At this stage, the precipitate formed in the system is carefully removed. The mixture 2 is then filtered to furthermore remove undesired residues of lead oxide. The so obtained mixture 2 is then blended with mixture 1 (the procedure is similar to the one reported in [26]) and the complete final mixture is kept under magnetic stirring for further 36 h in dark.

### 2.3. Experimental Set-Ups

Our standard cell is made by two microscope glasses separated by two 76 μm thick Mylar stripes. The cell is heated at around 60 degrees and the mixture is forced to enter by capillarity. After this, the sample, left for one hour at room temperature on the sample holder, is irradiated by two interfering continuum s-polarized laser beams at 457.9 nm (see Setup (A) in Figure S1 in Supporting Information Section). During the photo-polymerization, a phase separation process occurs between polymerized and unpolymerized regions of the mixture and, as a result, a one-dimensional (1D) holographic grating is permanently recorded inside the interfering region. This region has a diameter of 5 mm. The used writing power is 150 mW per beam. A low power 632.8 nm He-Ne laser positioned at the Bragg diffraction angle is used to detect the grating formation. After three minutes, the grating is completely recorded. To ensure a complete photo-polymerization of the spot area, the total irradiation time is set at 10 min. The sample is post-polymerized for one minute using a U.V. incoherent lamp (P = 0.5 W, λ = 365 nm). The spectral angular analysis of the diffracted wavelengths is performed by illuminating the grating, placed on a motorized goniometer, through a He-Ne laser. Data are acquired for each value of the incident angle (see experimental Setup (B) in Figure S2 in the Supporting Information Section). Finally, a camera provided with a free software (Pendent Drop ImageJ plugin) was used to perform measurements on droplet’s surface tension changes of mixture 2. The same camera was used to detect the droplet’s displacement under irradiation.

## 3. Theory and Experiments

The diffraction efficiency of a transmission phase grating is given by the well-known Kogelnik theory [27]. Accordingly, the diffraction efficiency of a one-dimensional transmission phase grating can be written as:

(1) $$\eta \left(\nu ,\xi \right)=e^{-\frac{\alpha d}{\cos\theta }}\frac{{sin\left(\sqrt{\left(\nu^{2}+\xi^{2}\right)}\right)}^{2}}{1+\frac{\xi^{2}}{\nu^{2}}}$$

with coupling and detuning parameters, respectively defined as:

(2) $$\nu =\frac{\pi \cdot \delta n\cdot d}{\lambda \cdot cos\theta }$$

(3) $$\xi =\Delta \theta \cdot \beta \cdot d\cdot sin\theta_{0}$$

as δn is the induced refractive index variation, *d* the grating thickness, λ the reading wavelength in the free space, θ the angle of incidence, $\theta_{0}$ the Bragg angle, α the distributed absorption coefficient, and *n* the average refractive index of the medium, and β=2πn/λ.

The parameter Δθ in Equation (3) describes the de-phasing term appearing when λ or θ are varied.

**Figure 1:**
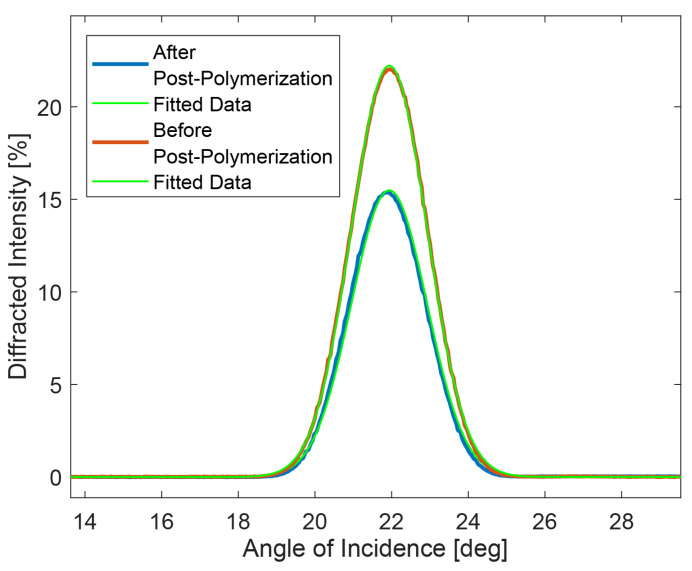
Effects of post-polymerization on the diffraction efficiency of the transmission grating written in the photomobile polymer material.

The results obtained from the angular analysis are reported in Figure 1, which shows the diffraction efficiency for the vertical reading polarization as a function of the incidence angle. The two curves represent the diffraction efficiency before and after the post-photopolymerization process. The two continuous lines represent the theoretical data fits obtained by using Equation (1). As we can clearly see, the post-photo-polymerization process reduces the diffraction efficiency of about 30%. The post-polymerization process is indeed needed in order to obtain a useful free standing H-PMP. This phenomenon can be attributed to the photophobic nature of the material, at the basis of the mechanism of the motion of the photomobile polymer film [26]. When a powerful high energy light impinges on the grating, the internal unpolymerized photophobic material tends to escape from the solid polymeric grating. Macroscopically, if continuing to impinge on the system with the same power, the system could partially collapse, due to the thin thickness of the acrylate walls (around 250 nm × 75 µm). The values of optical index contrast reduce, and as a consequence, the diffraction efficiency decreases. Data fit shows an excellent agreement between the theoretical expression reported in Equation (1) and the experimental data and allows the determination of the grating refractive index modulation $\delta n_{1}∼2.4\times 10^{-3}$, $\delta n_{2}∼3.4\times 10^{-3}$, pitch Λ = 512.8 nm and thickness *d* = 77.98 μm. Here, $\delta n_{1}$ represents the refractive index modulation before post-photopolymerization, and $\delta n_{2}$ the refractive index after post-photopolymerization. The data have been corrected for angle- and polarization-dependent Fresnel refraction.

**Figure 2:**
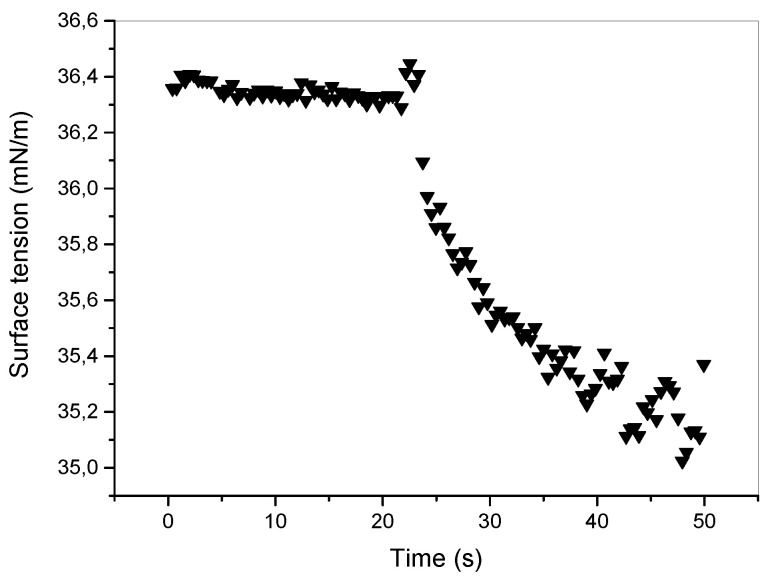
Surface tension as function of time under light irradiation (λ = 405 nm; P = 25 mW).

**Figure 3:**
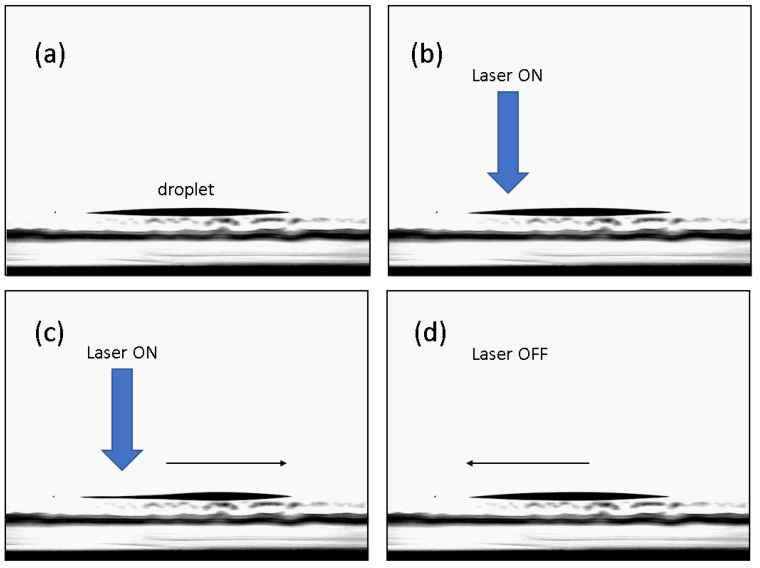
A droplet of unpolymerized material on a glass surface (**a**). Under laser irradiation, the droplet tends to escape away from the irradiated area (**b**,**c**). When the laser is switched off, the material returns in its original position (**d**).

Concerning the photophobicity nature of the unpolymerized part of the H-PMP film, we underline how thermal effects play the most important role in the entire process. One of the first experimental observations made on a free pendant small droplet of unpolymerized PMP mixture was indeed its surface tension reduction (due to the increase in temperature), and consequently, its volume increases under laser irradiation (see Figure 2). Figure 2 represents the surface Tension measurements under illumination of a pendent droplet of NVP/4-AP-ox. When illuminating a NVP/4-AP-ox droplet by an unfocused low-power low-coherent laser light (λ = 405 nm; light density ≈ 0.130 W cm${}^{-2}$) its surface tension is almost instantaneously reduced from ≈36.3 to 35.4 mN m${}^{-1}$. Furthermore, we observed the photophobic nature of this material when a laser-light impinged along the vertical direction on a thick droplet placed on a horizontal surface. The droplet escaped rapidly from the irradiated area and returned when irradiation is turned-off. We think that this behavior explains what happens inside the gratings’ channels. When the unpolymerized H-PMP mixture escapes along the polymerized channels, the mechanical properties of the film change and a macroscopic bending are observed. At the end of irradiation, the mixture returns inside the channels and the initial condition are restored [21]. Figure 3 is a clear evidence of the mobility of the unpolymerized-PMP-mixture under irradiation.

**Figure 4:**
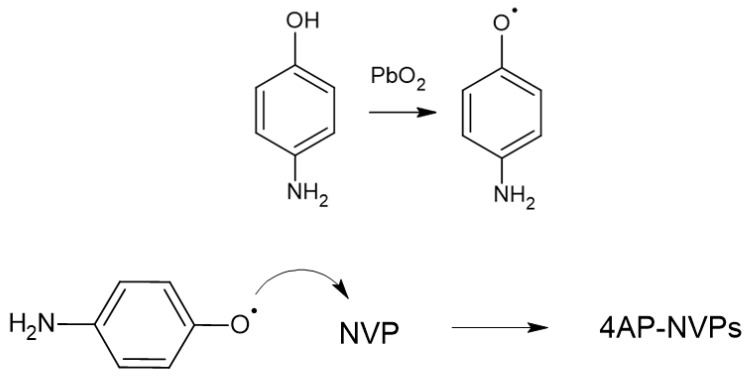
Scheme of the hypothesized reaction mechanism of NVP and 4-AP in oxidative conditions: formation of the phenoxy radical by PbO${}_{2}$ on 4-AP; attack of the phenoxy radical on NVP and oligomer formation.

Three experimental observations are here added: (1) the reaction of oxidation of para-amino-phenol by PbO_2_ could take place even in solid; (2) NVP in presence of PbO_2_ doesn’t seem to react even leaving the suspension of granular 4-AP in NVP for three days, in our experimental conditions. Furthermore, such a suspension does not seem to have any connection with photophobic phenomena; (3) when adding PbO${}_{2}$ to 4-AP in NVP, immediately, the suspension turns on a red dark colored solution and has photophobic properties. Reasonably, amino-phenoxyl-molecule could be generated by hydrogen abstraction on the hydroxyl group of the para-amino-phenol. As a consequence, phenoxyl-radical could be responsible for the further reactivity of 4-AP and NVP, since to the first intitial production of the hydroxyl radical, follows its attack to NVP-vinyl group (see Figure 4), probably bringing possible oligomers of NVP (see FT-IR of Ref. [26]). Furthermore, we cannot exclude a coordination by lead oxide in the system. All these hypotheses are under investigation. This material, however, is actually at the basis of the photophobic nature of the H-PMP evidenced in Figure 3.

## 4. Conclusions

In conclusion, we report the angular analysis of one-dimensional holographic transmission phase grating, written on a novel class of photomobile composite polymers: H-PMP material. The experimental data show the presence of a volume phase grating with a pitch Λ = 512.8 nm and a maximum diffraction efficiency of about 22%, at around 22°, which decreases by 30% after post-polymerization. An explanation of the working mechanism, based on the photophobic nature of the unpolymerized part of the mixture and surface tension changes, is proposed.

## Data Availability

Data are available from the authors.

## Supplementary Materials

The following are available online at https://www.mdpi.com/article/10.3390/mi13020297/s1. Figure S1: experimental Setup (A), Figure S2: experimental Setup (B).
