# Supplementary material for: Light-Induced Dynamic Holography
Source: Micromachines (Basel). 2022 Feb 14;13(2):297. doi: 10.3390/mi13020297 (PMC8877614; doi:10.3390/mi13020297)
Supplement: Supplementary file 1 [file micromachines-13-00297-s001.zip › micromachines-1561074-supplementary.pdf]

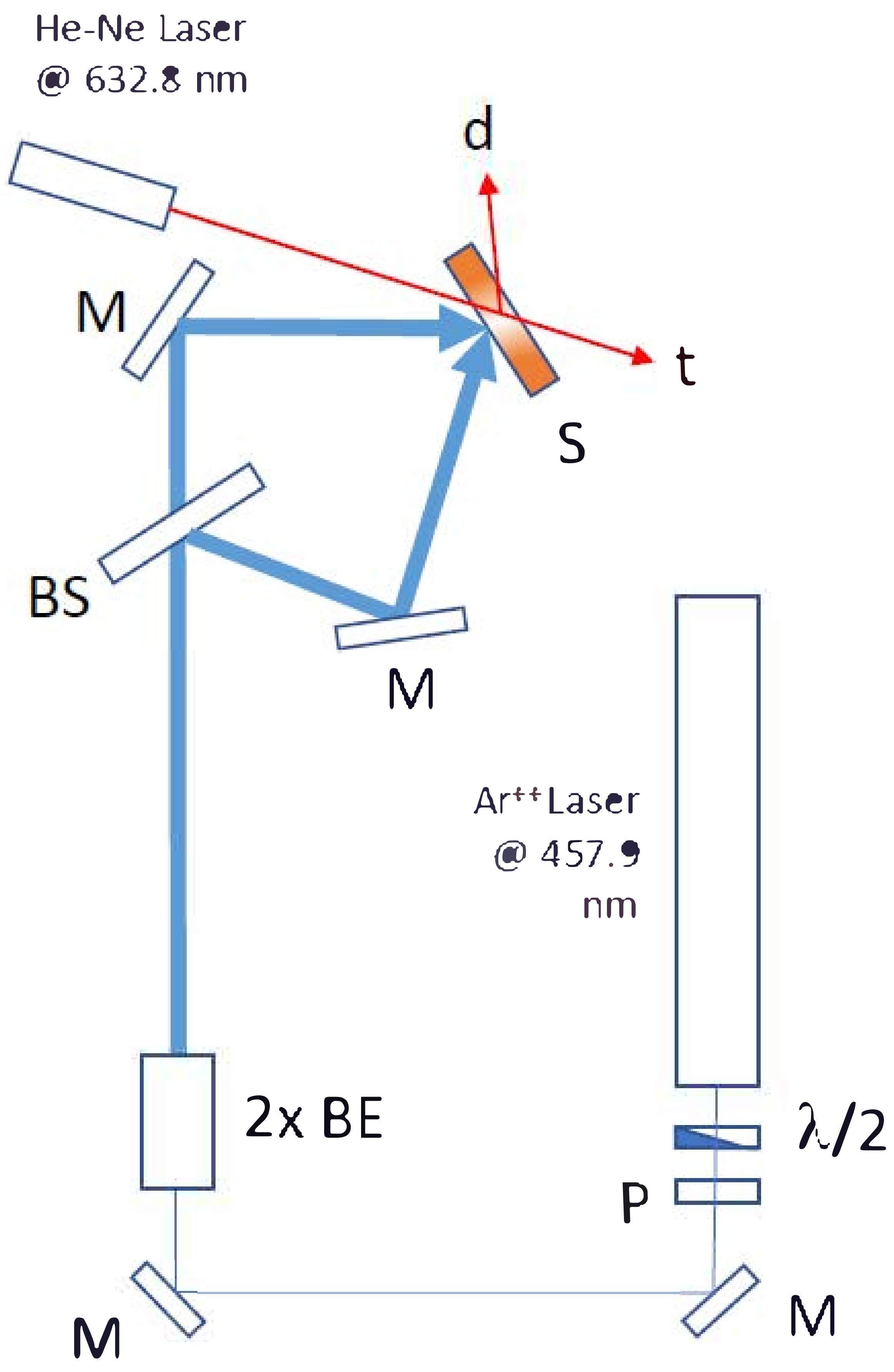

Setup (A)

λ/2=half wavelenght plate; P= polarizer; M=Mirror; 2x BE=2x Beam Expander; BS= Beam Splitter; S=sample; d and t= diffracted and transmitted beams respectively

Figure S1

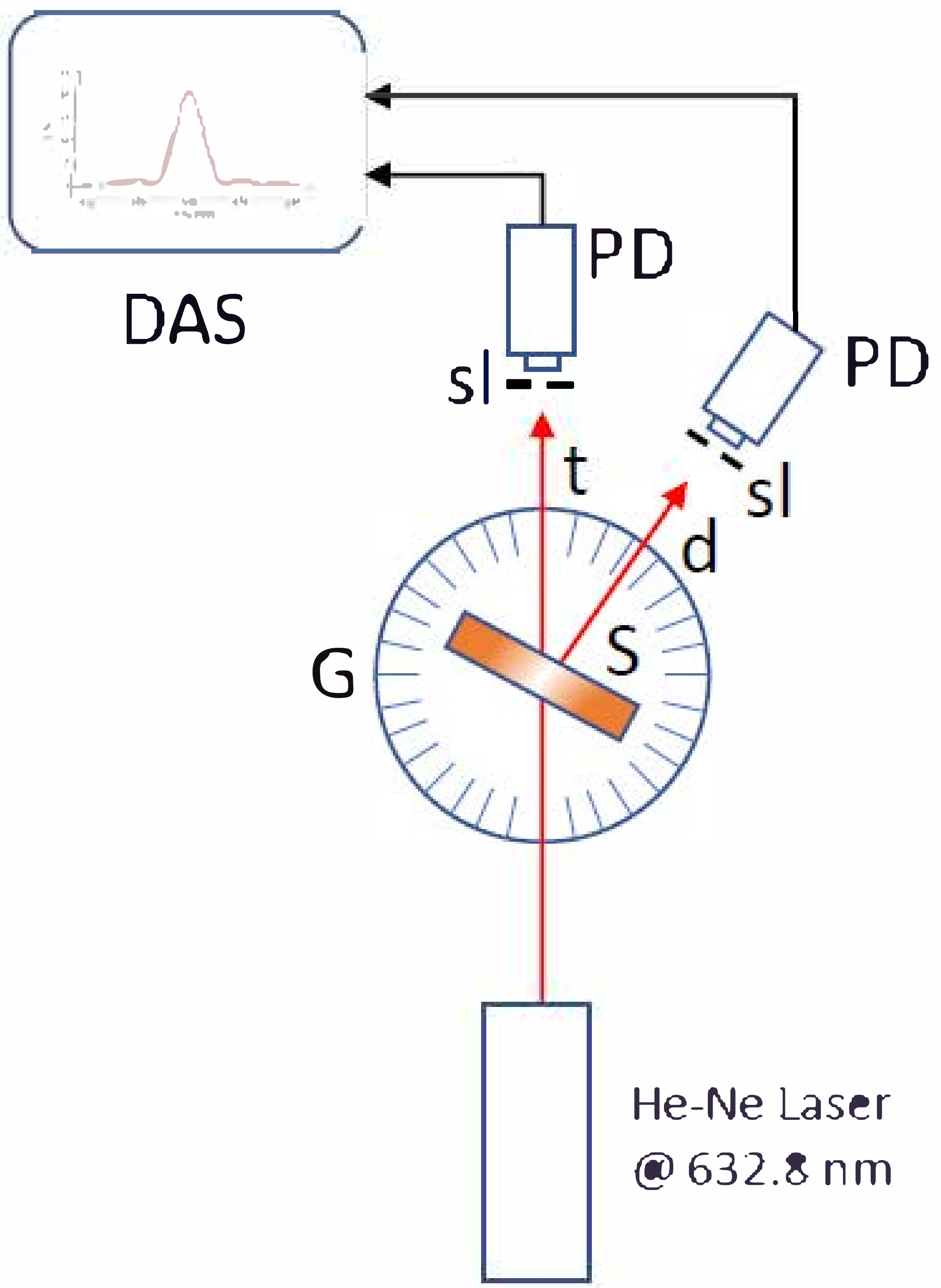

Setup (B)

G=Computer controlled Goniometer; S=sample; d and t= diffracted and transmitted beams; PD=Photo Detector; sl=slit; DAS=Data Acquisition System

Figure S2
